# Fabrication and Characterization of Bioactive Gelatin–Alginate–Bioactive Glass Composite Coatings on Porous Titanium Substrates

**DOI:** 10.1021/acsami.2c01241

**Published:** 2022-03-22

**Authors:** Belen Begines, Cristina Arevalo, Carlos Romero, Zoya Hadzhieva, Aldo R. Boccaccini, Yadir Torres

**Affiliations:** † Departamento de Química Orgánica y Farmacéutica, Facultad de Farmacia, Universidad de Sevilla, c/ Profesor García González 2, Seville 41012, Spain; ‡ Departamento de Ingeniería y Ciencia de los Materiales y del Transporte, Escuela Politécnica Superior, c/ Virgen de África 7, Seville 41011, Spain; § Department of Materials Science and Engineering and Chemical Engineering, Universidad Carlos III de Madrid, Av. de la Universidad 30, Leganés, Madrid 28911, Spain; ∥ Institute of Biomaterials, Department of Materials Science and Engineering, University of Erlangen-Nuremberg, Cauerstraße 6, Erlangen 91058, Germany

**Keywords:** porous titanium, biopolymer composites, tribomechanical behavior, osteochondral defects, bioactive coating

## Abstract

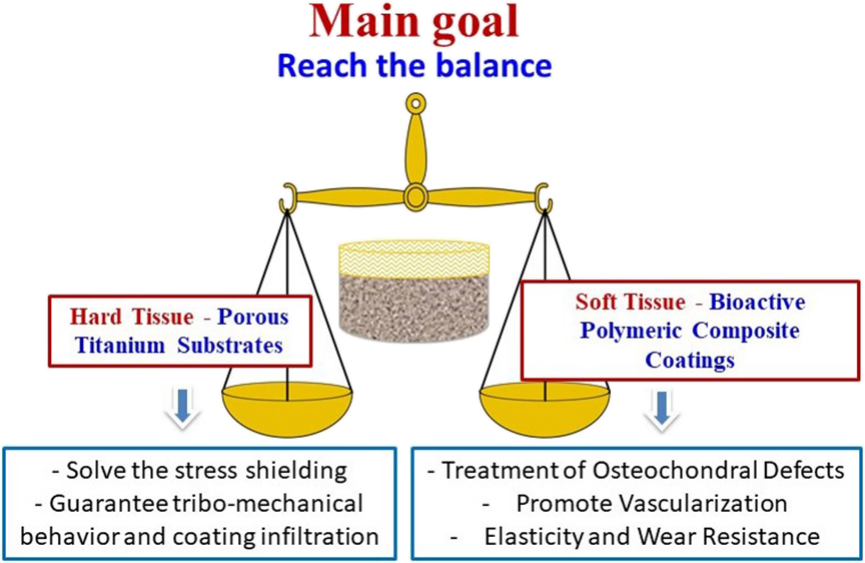

In this research work, the fabrication of biphasic composite implants has been investigated. Porous, commercially available pure Ti (50 vol % porosity and pore distributions of 100–200, 250–355, and 355–500 μm) has been used as a cortical bone replacement, while different composites based on a polymer blend (gelatin and alginate) and bioactive glass (BG) 45S5 have been applied as a soft layer for cartilage tissues. The microstructure, degradation rates, biofunctionality, and wear behavior of the different composites were analyzed to find the best possible coating. Experiments demonstrated the best micromechanical balance for the substrate containing 200–355 μm size range distribution. In addition, although the coating prepared from alginate presented a lower mass loss, the composite containing 50% alginate and 50% gelatin showed a higher elastic recovery, which entails that this type of coating could replicate the functions of the soft tissue in areas of the joints. Therefore, results revealed that the combinations of porous commercially pure Ti and composites prepared from alginate/gelatin/45S5 BG are candidates for the fabrication of biphasic implants not only for the treatment of osteochondral defects but also potentially for any other diseases affecting simultaneously hard and soft tissues.

## 1 Introduction

According to the World Health Organization (WHO), musculoskeletal disorders consist of a group of more than 150 conditions affecting the locomotor system of approximately 1.71 billion people all over the world.^1^ The increment in the global population and the life expectancy is leading to a rapid increase of this type of disease that affects all ages and entails different grades of disability,^2−4^ from short-lived to chronic conditions. Among the disorders that may affect the musculoskeletal system, the most numerous ones are those affecting bones and joints, such as osteoporosis, osteochondral defects, osteoarthritis, or rheumatoid arthritis, obviously in addition to accident-derived injuries. Independently of the disorder origin, the need for implants as bone and/or articular replacements is becoming a public health issue nowadays. For example, only in the United States, more than 1 million arthroplasties are performed every year for the total replacement of the knee or the hip, and the number of such interventions is expected to increase to 4 million by 2030.^5,6^ In particular, diseases affecting both the bone and the cartilage, such as osteochondral defects, require the development of therapies based on the replacement of both tissues.^7−10^ In fact, different treatments based on autograft or allograft transplantations or bioengineered tissues have been already applied, but they only address the articular cartilage.^11^ Therefore, the use of multiphasic implants has been investigated in the past years to substitute the bone and cartilage tissues in one single surgery.^12,13^ Such implants are usually constituted of a rigid section to replace the bone and a soft coating mimicking the cartilage tissue,^14−18^ although in some cases, a third layer is added as the interface between the rigid and the soft zones.^19−22^

Nowadays, independently of their fabrication method, most of the biphasic implants proposed for the treatment of osteochondral defects are based on polymeric materials. Thus, polymers such as collagen I,^22^ gelatin,^17^ silk fibroin,^16^ polyglycolide,^18^ or polycaprolactone^23^ have been already investigated as bone substitutes. However, just a very reduced number of research works have considered the application of metals as the rigid section of the implant,^11,24,25^ considering their suitable mechanical properties for bone tissue replacement. One of the most commonly used metals in prosthesic applications is Ti, and its corresponding alloys, due to its exceptional biocompatibility, mechanical characteristics, and excellent corrosion resistance.^26−28^ However, the high Young’s modulus of Ti and Ti alloys is an important limitation, causing a stress shielding phenomenon,^29^ with consequent reduction in the surrounding bone density. To reduce the difference in the Young’s modulus between the implant and the bone and to modulate the substrate stiffness, the introduction of porosity in the construct has been attempted. Although generation of pores in metallic implants has been investigated by means of additive manufacturing, powder metallurgy, or foaming techniques,^30−33^ the space holder fabrication procedure, based on powder metallurgy, has gained high relevance due to its versatility, reliability, and low cost.^34−38^

On the other hand, different approaches have been tested to develop new biomaterials as cartilage tissue replacement,^12,39−42^ in particular, and tissue engineering, in general.^43−45^ Polymers and polymeric composites are the most commonly used materials considered for this application^46−48^ due to their biocompatibility and tunable properties,^49−51^ which enable development of their chemical structure or composition according to the final aim. In this sense, among naturally occurring polymers, gelatin and alginate stand out for the possibility of tuning their mechanical properties thanks to the degree of cross-linking.^52,53^ Their biocompatibility have been widely investigated in different *in vitro* and *in vivo* experiments for diverse tissues,^54−57^ including the bone and the cartilage.^58−62^ In addition, they form hydrogels that allow the easy preparation of composites,^63−65^ for example, bioactive glasses,^45,66^ for the improvement of their osseointegration capacity.^67,68^

To our knowledge, apart from the authors of this manuscript, the only research work proposing a biphasic implant based on porous Ti was carried out by Duan et al.^11^ Thus, the main objective of the present research work is the development of biphasic implants intended for the treatment of osteochondral defects. The use of porous Ti to substitute the cortical bone tissue is proposed to reduce the stress shielding phenomenon. On the other hand, based on our previous work,^25^ a combination of gelatin and alginate incorporating 45S5 BG is presented as the material layer facing the cartilage tissue. Finally, the influence of the substrate porosity and the chemical composition of the polymeric composites on the tribomechanical and biofunctional behavior of the biphasic partial implants will be evaluated.

## 2 Materials and Methods

**Figure 1:**
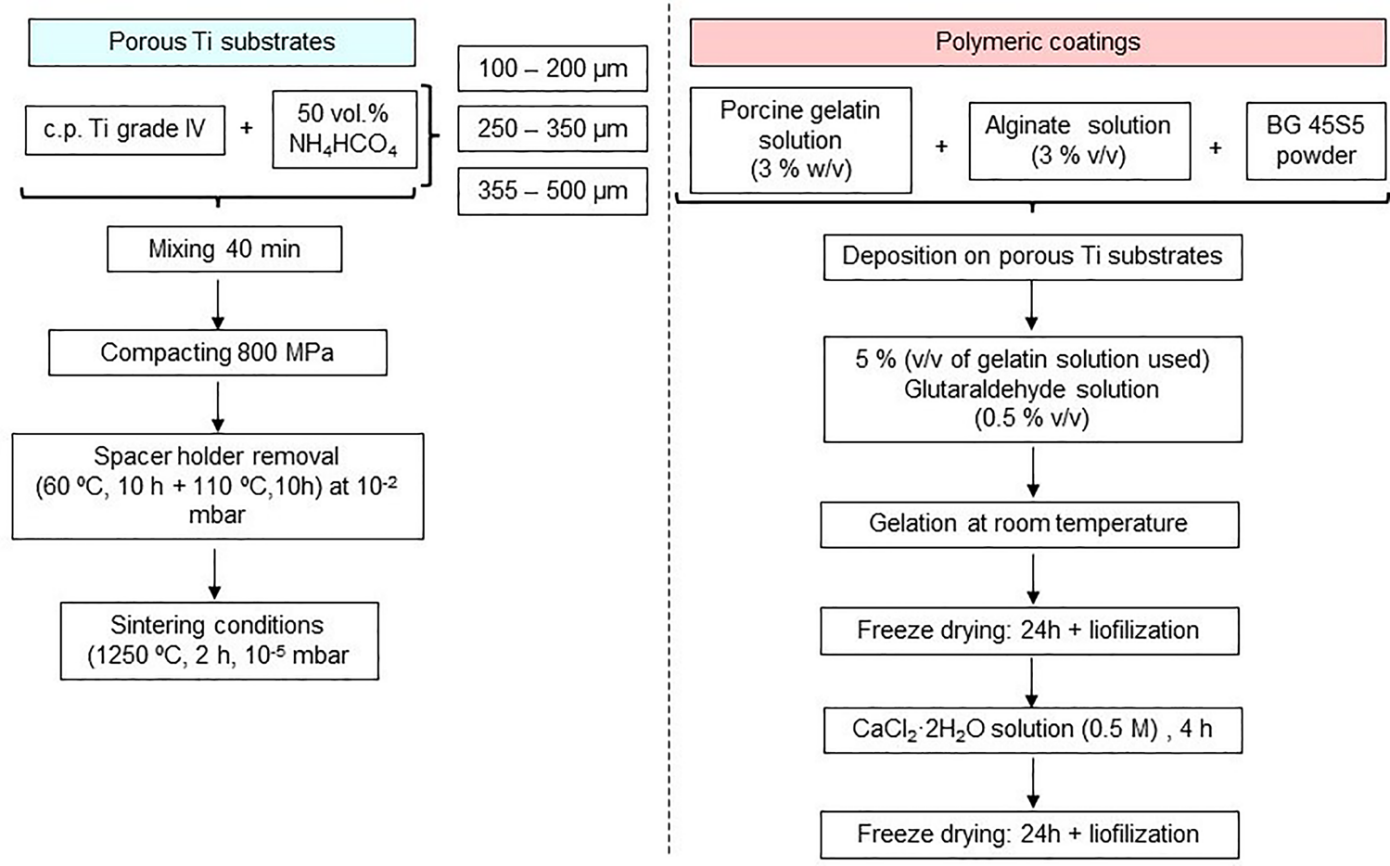
Schematic of the fabrication process of biphasic coated substrates.

The protocols to obtain the porous titanium substrates and the bioactive gelatin–alginate–BG composite coatings are summarized in Figure 1, while the fabrication and the characterization details are described in the next sections.

### 2.1 Processing of Porous Substrates and Polymeric Coatings

Commercially pure (c.p.) titanium powder, grade IV (SE-JONG Materials Co., Ltd.), was mixed with 50 vol % ammonium bicarbonate (NH_4_HCO_3_) with different particle size ranges (100–200, 200–355, and 355–500 μm) (Cymit Química S.L.). The mixtures were pressed at 800 MPa, and the spacer was removed using two thermal cycles, both applying a low vacuum (10^–2^ mbar): the first one at 60 °C for 10 h followed by another treatment at 110 °C for 12 h. Afterward, the porous green samples were sintered in a molybdenum chamber furnace, at 1250 °C for 2 h and high vacuum conditions (∼10^–5^ mbar). Finally, before depositing the coatings, the surface of the titanium substrates (discs of 12 mm diameter and 3 mm height) was carefully ground and polished with magnesium oxide and hydrogen peroxide, to preserve the porosity fraction, size, and morphology of the pores inherent to the spacer.

In this work, different biopolymers were deposited, combining gelatin powder from porcine skin and sodium alginate (both purchased from Sigma-Aldrich), after considering the degradability, bioactivity, and processing conditions. After selecting the polymers, a route for the preparation of the solutions and the cross-linking of the polymers was fixed, as they need to have a longer degradation time. Figure 1 shows the sequence for obtaining polymeric coatings and composite materials.

For the preparation of the gelatin soft phase, an aqueous solution of 0.03 g/mL gelatin was assembled by stirring at 60 °C for 1 h, until it was completely dissolved. The cross-linking solution consisted of 0.5 vol % (v/v) glutaraldehyde solution, prepared from a concentrated solution (50 vol % glutaraldehyde). The cross-linking agent was added to the gelatin solution in a proportion of 0.05 mL of glutaraldehyde solution per milliliter of gelatin solution. Concerning the alginate phase, an aqueous solution was prepared with 0.03 g/mL sodium alginate under stirring at room temperature for 1.5 h until full dissolution. The cross-linking process of the alginate was done after freeze-drying; the sample was immersed for 4 h in a 0.5 M CaCl_2_·2H_2_O aqueous solution and freeze-dried again.

Then, different compositions of the polymeric soft phase were prepared, always maintaining the concentration of the polymer in the solution at 0.03 g/mL, to produce biphasic coatings. When preparing the polymer blend solutions, first, the alginate solution was added dropwise in a beaker and next, the uncross-linked gelatin solution. Both solutions were produced in the proportions shown in Table 1. After stirring for 10 min for homogenization, the cross-linking solution was added according to the content of gelatin. Once the solution was assembled, it was placed dropwise onto a plate and left gelling at room temperature overnight. Subsequently, the samples were put in a freezer at −20 °C. After the freezing process, they were put into a freeze-dryer for 24 h. Later, the samples containing alginate were cross-linked according to the process described above.

**Table 1:** Polymeric Compositions Used

alginate (%)	100	75	50	25	0
gelatin (%)	0	25	50	75	100
blend name	A	3A1G	1A1G	1A3G	G

Finally, as the intention was also to increase the bioactivity of the soft phase, bioactive glass (45S5 BG, composition: 24.5 wt % Na_2_O, 24.5 wt % CaO, 45 wt % SiO_2_, and 6 wt % P_2_O_5_) was added to the polymer blend. The BG powder presented a *d*_50_ of 4.5 μm, and it was received in an amorphous (noncrystalline) form. Several proportions of the polymer/BG were studied, using only alginate and only gelatin as polymers. The goal was to reach a uniform distribution with no sedimentation of BG particles. This was achieved just when alginate was employed at a concentration of 30 mg/mL and the BG content was 5% of the total weight (w/w) (1.58 mg/mL) of the final composite. Two aqueous solutions, one of gelatin and the other of alginate, were prepared, with a polymer concentration of 30 mg/mL. The gelatin solution was stirred at 60 °C for 1 h and the alginate one at room temperature for 1.5 h. Then, they were mixed in the proportions shown in Figure 1, and BG powder was added at a concentration of 1.58 mg/mL, all under constant stirring. After that, the cross-linking solution (0.5 vol % glutaraldehyde in distilled water) was added in a proportion of 0.05 relative to the gelatin solution content by volume. The solution was placed in well plates and left gelling overnight. In the case of preparing the biphasic implants, the composite suspension was poured onto the substrate that was previously put into a heat shrink tube to avoid leaking of the suspension. Then, the samples were inserted in a freezer at −20 °C and completely frozen and freeze-dried for 24 h. To cross-link the alginate, the samples were immersed in 0.5 M CaCl_2_·2H_2_O aqueous solution for 4 h and then freeze-dried again.

### 2.2 Microstructural, Biofunctional, and Tribomechanical Characterization of the Coated Porous Substrates

The density and total and interconnected porosity of the titanium substrates and polymeric coatings were determined by the Archimedes’ method, carefully cutting small cubes of approximately 27 mm^3^ in the case of the gelatinous composites. The morphology, size, and mean diameter of the pores of the substrates and the coatings were evaluated, using optical images (Nikon Epiphot microscope and Image-Pro Plus 6.2 software) and scanning electron microscopy (SEM) (Jeol JSM 6330F microscope). In particular, SEM images obtained on the cross sections of the studied materials allowed us to evaluate the homogeneity of the pore distribution, the degree of infiltration, and the adherence of gelatinous composites to the porous titanium discs.

On the other hand, the macromechanical behavior of the porous titanium substrates was evaluated in terms of yield strength (σ_y_) and dynamic Young’s modulus (*E*_d_), implementing the uniaxial compression test according to ASTM E9-09 (Instron 5505 universal testing machine)^69^ and an ultrasound technique (KRAUTKRAMER USM 35), respectively.

Then, a study of the swelling, degradation, and bioactivity of the biopolymeric composites was carried out. Swelling results were obtained by introducing the samples to phosphate-buffered solution (PBS) for 15 min, 30 min, 1 h, and 24 h and measuring their weight after the predetermined time points of immersion (*W_i_*). Samples were also weighted in the dry state (*W*_0_). The swelling (Sw) percentage was calculated according to eq 1.
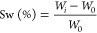
1

In order to study the degradation behavior, the immersion of the samples in PBS was continued for up to 14 days to evaluate if the composites remained undissolved and stable. Also, the samples were immersed in a simulated body fluid (SBF) for 3, 7, and 14 days to assess their bioactive behavior. After these periods, the samples were removed from the buffer solution, washed in distilled water for 1 or 2 min, and freeze-dried. The samples were then characterized by SEM imaging, energy-dispersive X-ray spectrometry (EDX), and Fourier transform infrared spectroscopy (FTIR) to look for hydroxyapatite formation (Ca/P ratio, elemental composition, and typical hydroxyapatite bonds).

Finally, since the polymeric samples were not completely flat, their surface (top view) was prepared by carefully cutting a thin sheet of the outer layer—removing only the material required to obtain a flat and homogeneous area, just to ensure a completely flat surface—and/or carefully polishing it by hand, to avoid damage of the surface or interfering in its microstructure and simultaneously to obtain a flat area that would allow a correct evaluation of the tribological behavior. In this context, although the wear resistance results could be affected by the presence of the substrate depending on the elastic deformation, the thickness of the composite layer will be high enough to simulate the cartilage tissue and avoid the substrate effect. The wear resistance of the coatings was studied by a ball-on-disc test (Microtest MT/30/NI tribometer). The studies were carried out at room temperature, with a radius of 1 mm and with 6 mm diameter alumina balls. A dead load of 1 N and a rotation speed of 300 rpm (31.4 mm/s) were applied, and the distance traveled was between 5 and 15 m. Some additional experiments using more critical wear conditions (greater load and distance) were also carried out. These experiments allowed us to rule out some of the studied coatings (excessive wear was observed). During the wear tests, the penetration depth was continuously recorded (LVDT vs distance). The results were analyzed in terms of the mass loss, wear kinetics (threshold and slope of the curve) of the biopolymers, and the sliding contact response that was given in terms of the coefficient of friction (COF). It was computed through the normal and tangential force, both measured by a strain gauge-type dynamometer. Also, absolute wear rates were calculated as follows (eq 2):
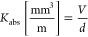
2where *V* is the volume of the worn material, estimated from the mass loss and the density of the composites measured via the Archimedes’ method, and *d* is the distance traveled during the test.

## 3 Results and Discussion

### 3.1 Fabrication and Characterization of Ti Substrates

**Figure 2:**
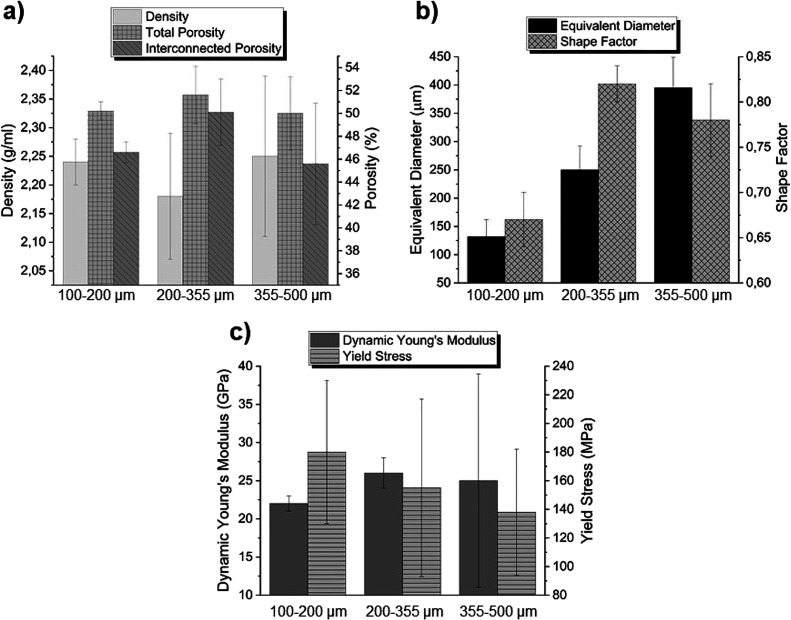
Microstructural characterization of porous c.p. Ti substrates: (a) density and total and interconnected porosity and (b) equivalent diameter and shape factor as well as mechanical behavior: (c) dynamic Young’s modulus (*E*_d_) and yield strength (σ_y_).

Details related to the protocols of characterization of the porosity and the mechanical behavior of the porous titanium substrates can be consulted in other previous works published by the authors of this investigation.^25,70,71^ In general, c.p. Ti substrates were produced following the space holder technique as depicted in Figure 1, considering a 50% spacer with three different particle size ranges: 100–200, 200–355, and 355–500 μm. The as-fabricated samples were characterized in terms of density and porosity (total and interconnected). As shown in Figure 2a, the density was very similar in all cases, ranging from 2.18 to 2.25 g/cm^3^. The total porosity of all discs ranged from 50 to 51.6%, close to the initial 50% space holder volume content. In addition, most of the pores were interconnected, showing an interconnected porosity from 45.6 to 50.1%, which is favorable for biopolymer infiltration. The equivalent diameter (Figure 2b), obtained by image analysis, showed values of 132, 250, and 395 μm for the three pore size distributions, respectively, with shape factors of 0.67, 0.82, and 0.78. Therefore, it could be concluded that the space holder technique is a very adequate technique to fabricate porous Ti substrates with elevated controlled porosity. Results depicted in Figure 2 indicate, in general terms, that (1) the total and interconnected porosity followed the same trend and (2) the values of interconnected porosity were relatively high; this fact, as well as pore sizes greater than 100 μm, favors the infiltration and adherence of the composites. In this context, the substrate with intermediate size distribution is the one that showed better adhesion of the coating, as it will be discussed later. (3) If the extreme particle size ranges are compared (100–200 vs 355–500 μm), for the same pore content (approx. 50 vol %), the pores were more isolated as the pore size increased. On the other hand, biomechanical characterization of substrates was conducted measuring their dynamic Young’s modulus (*E*_d_) and yield strength (σ_y_) (Figure 2c). In general, an inverse relationship was found between porosity and pore size and mechanical resistance of substrates: the higher the porosity, the lower the stiffness and therefore the lower the mechanical resistance.

### 3.2 Synthesis and Characterization of Polymeric Coatings

As mentioned above, in a previous research work published by our group,^25^ gelatin was tested as a potential cartilage tissue substitute in a biphasic implant. The main aim of this type of polymeric coating is the substitution of the cartilage functions in articulate joints. For these applications, the cartilage tissue must possess a good capacity for elastic deformation (pseudo-damping) during tribological solicitations, when damage caused by the applied wear conditions should be minimized. In addition, the coating porosity would allow its vascularization, while the presence of a BG would enhance the material’s bioactivity and therefore osseointegration. In this sense, polymeric blends of porcine gelatin and sodium alginate including BG 45S5 were investigated, using glutaraldehyde and Ca^2+^ ions as cross-linkers, respectively. Five different compositions were proposed, according to Table 1. They were named considering the proportion of each polymer in the final material. In this sense, the polymeric blend obtained from the use of 100% alginate was called A; the one prepared from 75% alginate and 25% gelatin was named 3A1G; the one synthetized from 50% alginate and 50% gelatin was 1A1G and so on. Although the use of glutaraldehyde has been defined as cytotoxic above certain levels, a previous study conducted by Lai demonstrated that concentrations lower than 0.03 mmol of glutaraldehyde per mg of polymer are well-tolerated by the human epithelial cells.^72^ In this research, the concentration of glutaraldehyde applied to the polymeric material was 10^–4^ mmol per mg of gelatin. In the case of the material prepared only with gelatin, the glutaraldehyde concentration is much lower than the limit established by Lai. In the rest of the polymer blends, the concentration is even lower. Therefore, the glutaraldehyde concentration used will not produce cytotoxicity.

**Figure 3:**
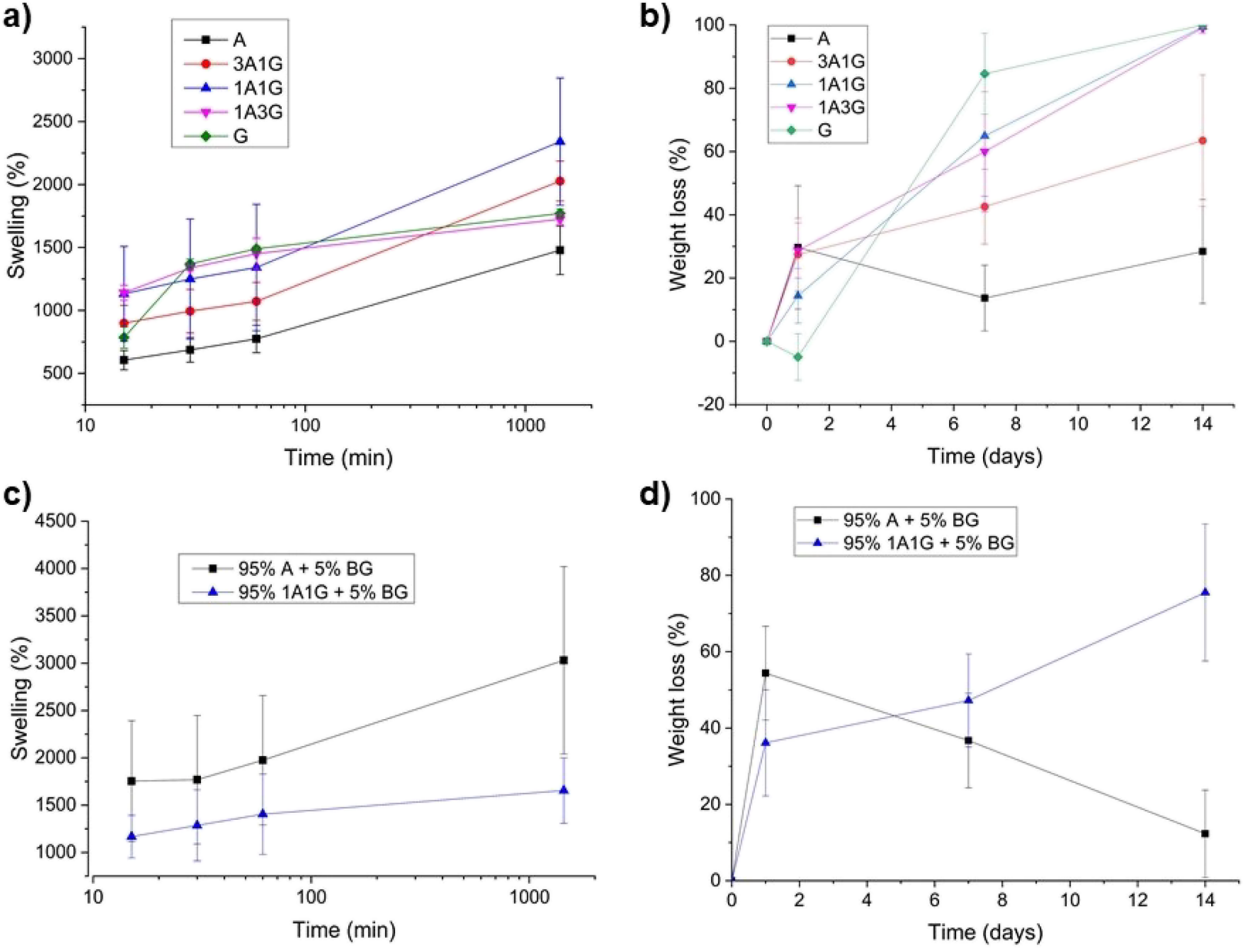
Characterization of polymeric coatings: (a,c) weight gain and (b,d) degradation rates of the different polymeric blends and composites.

To find the best candidate for the final aim among the five possibilities, a swelling test in PBS was conducted in the polymeric blends through the measurement of the weight gain in 24 h. The swelling behavior in the first 24 h of the different blends is depicted in Figure 3a. In general, all polymeric materials exhibited a significant capacity to swell in PBS, with weight gains ranging from approximately 15 to 23.5 times the weight of the original sample. It was noticeable that the gelatin showed a stronger hydrophilic behavior and swelled very fast but reached a plateau sooner, probably due to the beginning of its degradation. Moreover, there was no significant difference in the behavior of pure gelatin and a high gelatin content blend. This effect was not present in the blends containing a high percentage of alginate. This behavior could be explained by the nondegradability of alginate if the right enzyme, i.e., alginase, is not present in the medium,^52^ which maintains the structural integrity of the polymeric material. On the other hand, the presence of alginate reduced the swelling capacity of the blends, probably owing to the less hydrophilic character compared to gelatin and the different cross-linking type in each polymer. The polymeric chains of alginate remained closer after the ionic cross-linking, which hindered the PBS intake. Knowing these results, it was expected that the degradation of polymers with a high content of gelatin was more pronounced in PBS, as displayed in Figure 3b, since they were in contact with higher amounts of water, therefore improving the probability to suffer hydrolytic degradation. In fact, gelatin was almost completely degraded after 7 days of immersion in PBS. In addition and as previously mentioned, the alginate is not hydrolytically degradable^52^ under physiological conditions.

In light of these results, first, screening and selection of materials were conducted. In this sense, blends G and 1A3G, the ones with a higher content of gelatin, were dismissed for their fast degradation, mainly in the first hours of use. Therefore, the following experiments were continued with the polymeric materials A, 3A1G, and 1A1G.

**Figure 4:**
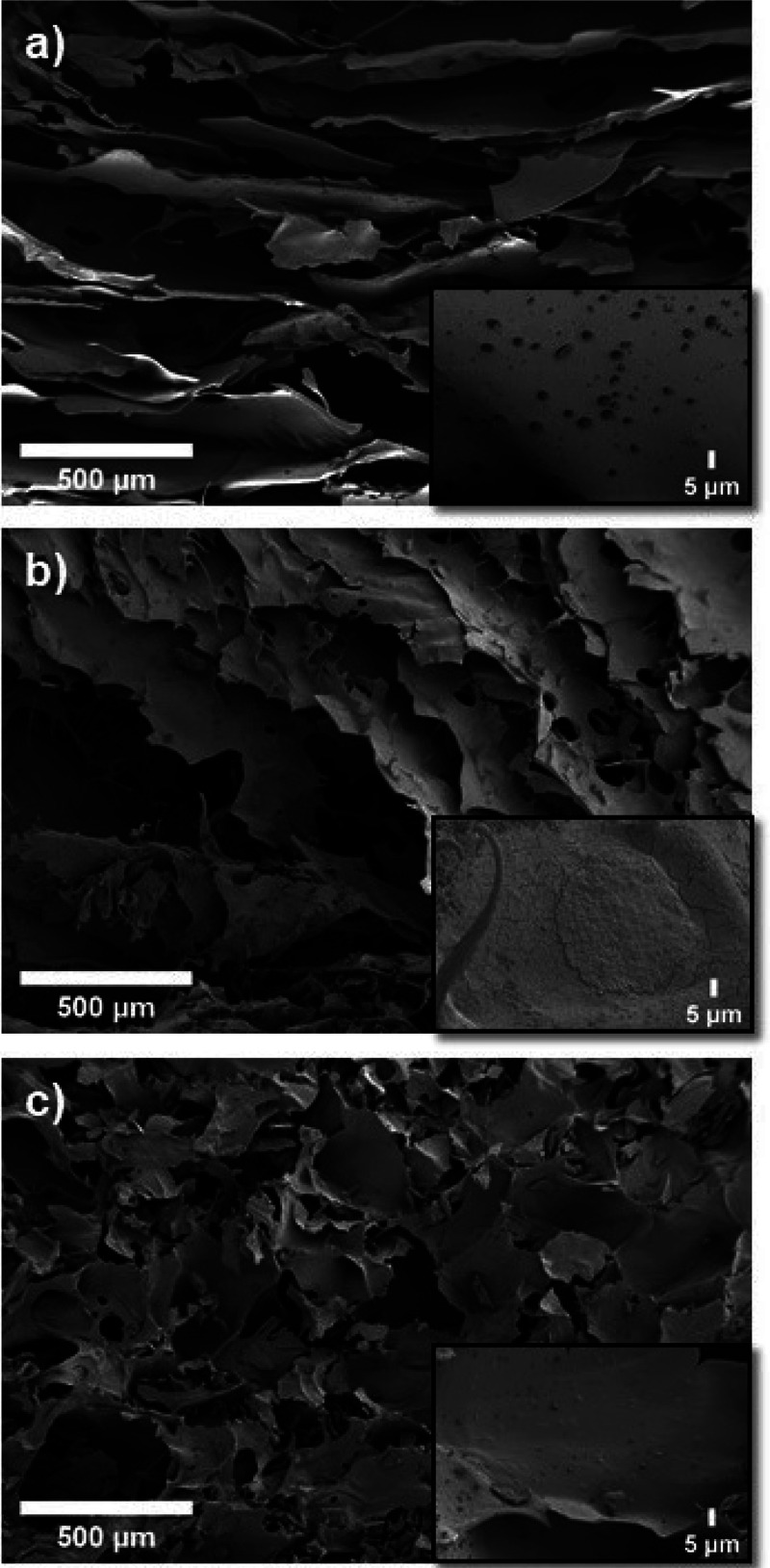
SEM micrographs of composites prepared from (a) A, (b) 3A1G, and (c) 1A1G, showing different pore structures. Insets: higher-magnification images.

To increase the bioactivity of the final material, by promoting the generation of hydroxyapatite, the addition of 45S5 BG was investigated. Thus, new polymer–ceramic composites were attempted by a homogeneous mixture of 95% (w/w) polymer (or polymeric blend) and 5% (w/w) bioactive glass, which was the maximum concentration of BG that avoided its sedimentation. In Figure 4, SEM micrographs of the three composites are displayed. They demonstrated that the microstructure of A presented a laminar form (Figure 4a), generating pores’ anisotropy, while the microstructure observed in 3A1G (Figure 4b) was more disordered and compact in the three directions of space. This effect was more pronounced in 1A1G (Figure 4c), presenting pore isotropy. This result indicates that the addition of alginate in the composite leads to a laminar structure and pore anisotropy that was sequentially modified to achieve pore isotropy with the addition of gelatin.

Using the microstructural characterization of the composites, another selection was carried out to reduce the number of samples for the final characterization. In this case, composites A and 1A1G were selected since they possessed a more differentiated pore structure, which could allow the investigation of the influence of the polymeric composite microstructure on its biofunctional and biomechanical behavior.

**Figure 5:**
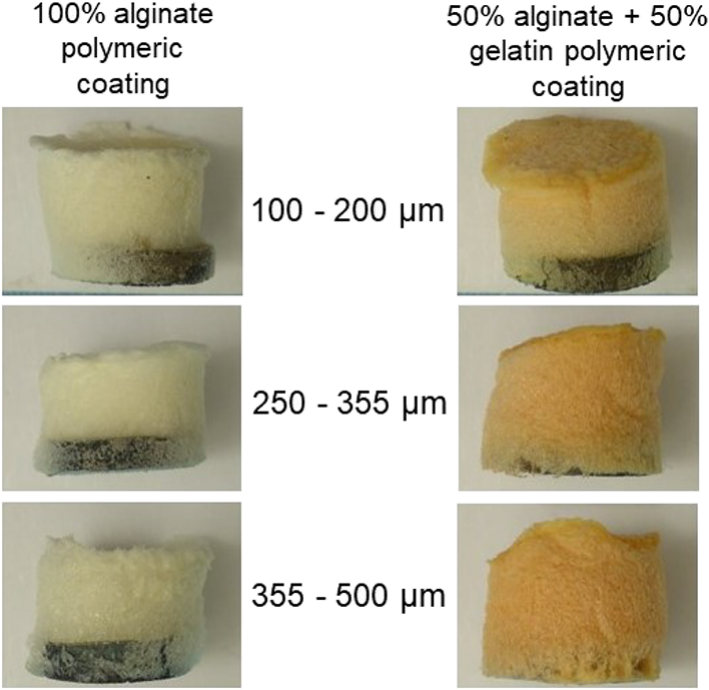
Optical images of the polymeric coatings onto metallic substrates. All samples contained 5 wt % BG 45S5.

**Figure 6:**
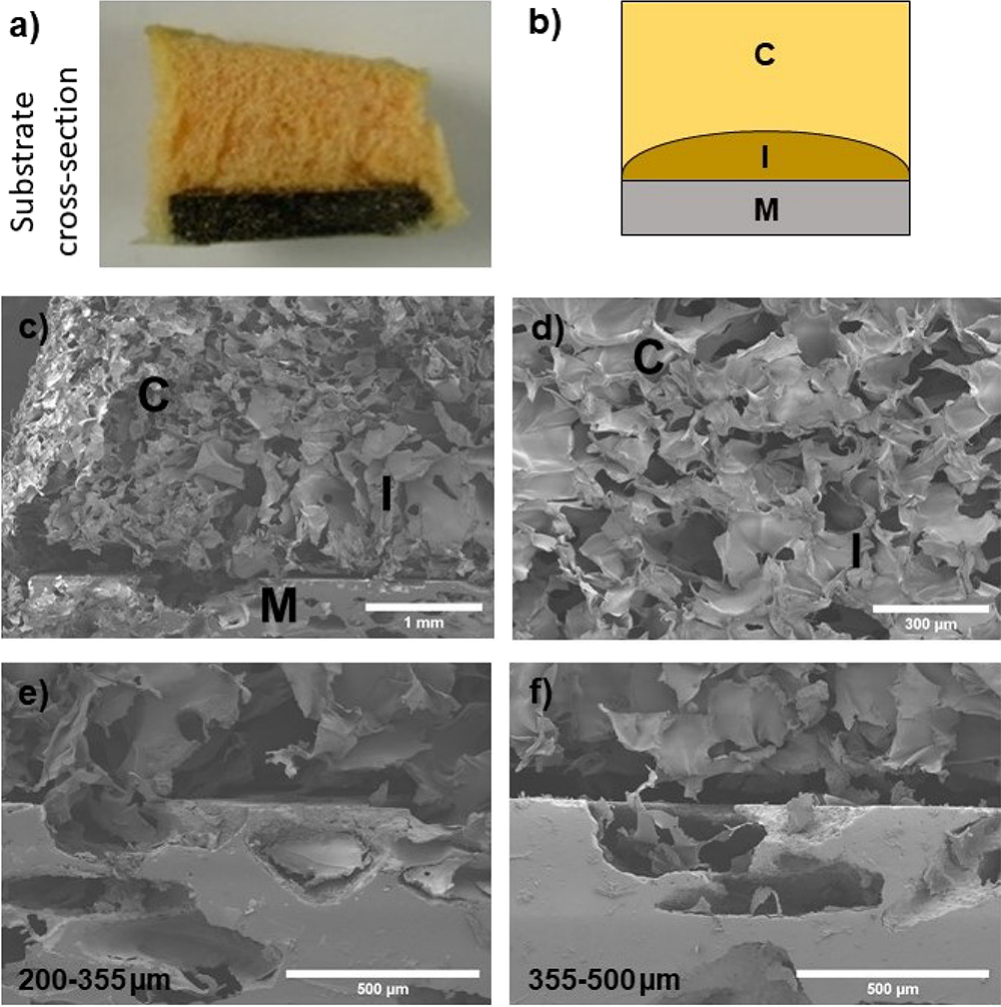
(a) Cross section of a substrate coated with the 1A1G composite. (b) Schematic of the coated substrate, where a composite interface (I) is illustrated between the composite coating (C) and the metallic sample (M). (c,d) SEM micrographs of the different areas, at two magnifications. 1A1G composite-coated substrates with two different pore size distributions: (e) 200–355 and (f) 355–500 μm.

The deposition of the selected composites on top of the different substrates was carried out, as depicted in Figure 5, generating a homogeneous coating as a soft foam, of white color in the case of the 100% alginate polymer and yellowish for the composite containing the polymeric blend. The faint yellow color shown by the gelatin-containing sample was due to the presence of glutaraldehyde applied as a cross-linker of this polymer.^25^ On the other hand, SEM pictures of the substrates’ cross section exposed the influence of the c.p. Ti substrate on the structure of both composites, independently of the pore size distribution. A schematic of this effect is displayed in Figure 6a,b, which represents material 1A1G. A D-shape composite area close to the polymer–metal interface (areas I in Figure 6c,d) was found, which seemed less porous than the rest of the composite (areas C in Figure 6c,d) due to the disc edge effect. Figure 6e,f proves the good infiltration and adherence of both composites onto the substrates’ pores. However, for pore size distributions of 100–200 and 355–500 μm, the polymeric coatings suffered from a slight detachment, an effect that was more accentuated for the composite 1A1G. The lower material adherence in extreme pore sizes could be related to a higher difficulty of infiltration for pores of 100–200 μm and a lower anchorage capacity of the coating in pore sizes of 355–500 μm due to a reduced number of anchor points. For substrates with similar pore contents, the increment of pore size entails a reduction in their number, and the pores are more isolated and therefore less interconnected. The higher interconnected porosity contained in samples with a pore size distribution of 200–355 μm resulted in a more efficient infiltration of the composite coatings.

**Figure 7:**
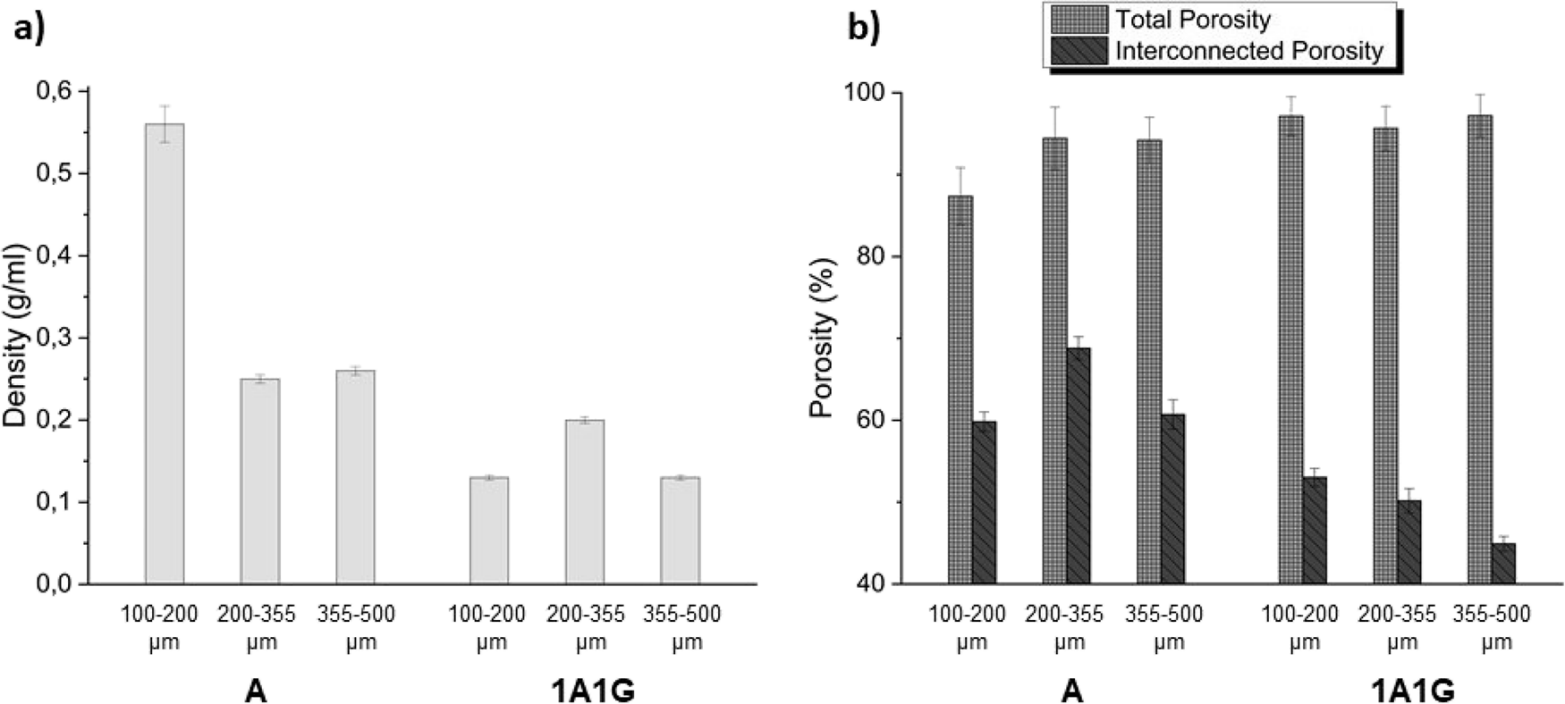
(a) Density and (b) total and interconnected porosity of the two tested materials: A and 1A1G. Note: the relative error of the density measurements varies between 2 and 4%.

These composites were also characterized in terms of density and porosity, total and interconnected, both estimated by the Archimedes’ method. The density values (Figure 7a) ranged from 0.13 to 0.56 g/mL (g/cm^3^). It should be noted that the density showed by the polymeric blend was lower than the value presented by the 100% alginate composite, probably due to the cross-linking of the gelatin, which induced a less compact material. This effect was expected as the differences in the cross-linkers for both polymers, glutaraldehyde for gelatin and calcium cations for alginate, are considerable. Therefore, comparing the results obtained from the density estimation (Figure 7a) and the microstructural characterization of composites carried out by SEM (Figure 4), it could be assumed that the increment of the gelatin content in the final composite led to the growth of pore isotropy that simultaneously entailed the decrease in the composite density. On the other hand, although all composites exhibited very high values of total porosity, the interconnected porosity was more elevated for the composite A. Meanwhile, the composite A that generated a coating with a higher interconnected porosity was the one deposited on substrates with 200–355 μm pore size distribution, which is in concordance with the higher values of interconnected porosity of this Ti sample. Therefore, the influence of the substrate’s pore size on the porosity of the coatings, where the coating tends to mimic the substrate porosity, was confirmed. The high total and interconnected porosity of the Ti substrates and polymeric composites will favor vascularization of the biphasic implant.

To check the effect of BG addition on the polymeric blends, swelling and degradation tests were carried out in A and 1A1G composites. Swelling tests (Figure 3c) showed that the capacity of 1A1G to absorb PBS was practically not affected by the addition of 5% BG 45S5 when compared with the counterpart blend (Figure 3a). However, composite A increases its swelling capacity, by roughly 3-fold, regarding the corresponding polymer. This could be explained by the fact that in 1A1G, as previously mentioned, the cross-linking of gelatin with glutaraldehyde was conducted prior to the gelling of alginate, and therefore, the degree of the first cross-linking governs the material capacity to absorb PBS. However, in the case of A, the cross-linking entirely comes from an ionic gelation with Ca^2+^. These ions may be partially trapped by the negatively charged oxygens (−O−) from the BG surfaces, which reduces the cross-linking degree of the alginate polymer and therefore increases its swelling capacity. Regarding the degradable behavior, in general terms, the addition of BG 45S5 to the polymers led to an enhanced biodegradability in the first hours of experiments (Figure 3d), possibly due to the effect of water interactions via the formed interfaces. The swelling/weight gain behavior of biopolymer composites containing bioreactive fillers, in the present case BG 45S5 particles, is complex. The different degradation mechanisms of the individual components, the possible change of pH following BG degradation, and the local (time-dependent) formation of hydroxyapatite (see the next section) due to the presence of BG must be considered, which depends on the fluid in which the test is carried out. The in-depth analysis of the (time-dependent) mechanisms involved during the degradation process is outside the scope of the present study and remains as a task for future investigations. In any case, the degradation of BGs has been demonstrated to be very low in similar systems. Thus, Zeimaran et al.^73^ showed a Ca^2+^ release of 9.6 × 10^–4^ mg of Ca^2+^/mg of composite. Therefore, the solubility of the BG could be considered as insignificant compared to the degradation of the polymer.

On the other hand, the biocompatibility of these polymeric materials has not been tested since, as previously mentioned, it has been widely proven by other authors in both *in vitro* and *in vivo* experiments for different tissues,^54−57^ including the bone and the cartilage.^58−62^

### 3.3 Bioactivity of Coated Substrates

**Figure 8:**
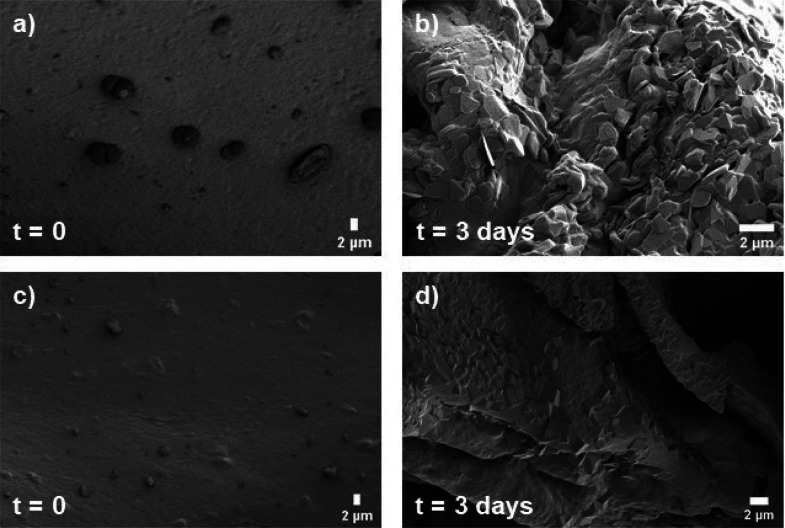
SEM micrographs of composites (a,b) A and (c,d) 1A1G before and after 3 days of immersion in the SBF.

**Figure 9:**
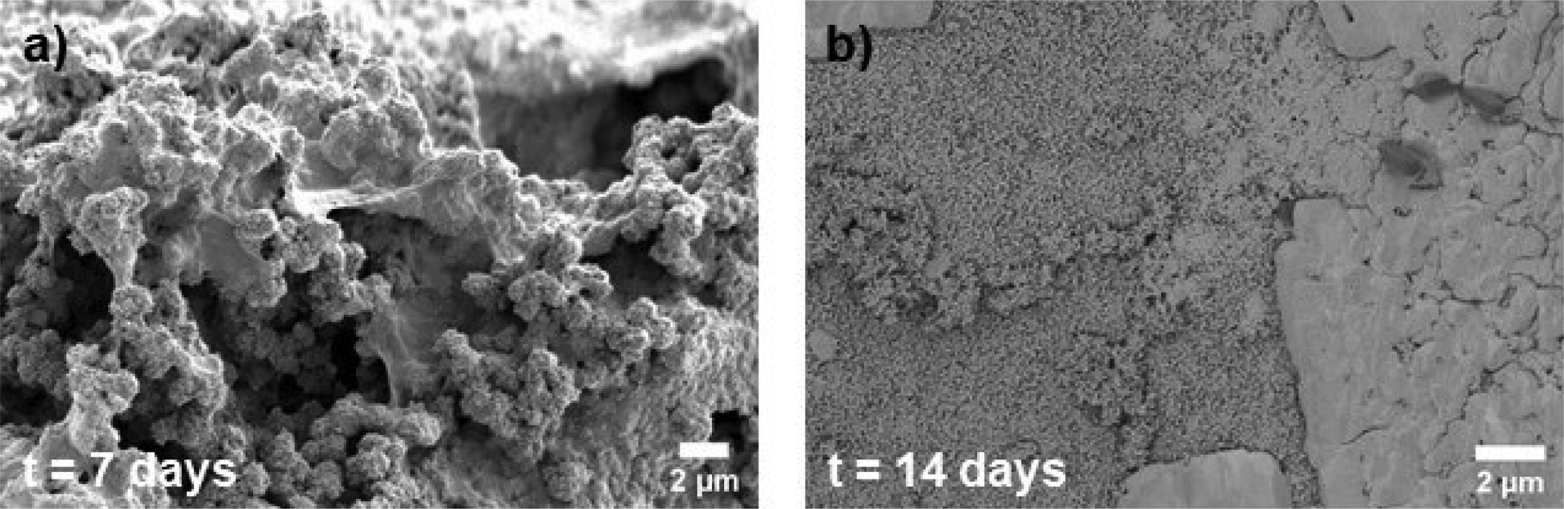
SEM images of composite A after (a) 7 and (b) 14 days of immersion in the SBF.

Samples fabricated with a space holder of 100–200 μm and coated with A and 1A1G composites were submerged in an SBF, and their surfaces were investigated by SEM after 3 days of immersion (Figure 8). No micrograph showed the presence of hydroxyapatite on the coatings. After 3 days in the SBF, only crystals of NaCl covering the original material were observed on both composites. Nevertheless, the bioactivity experiment was carried out to check if any variation on the coatings was perceived after longer periods of exposure to the SBF. A change in the morphology of the surface was monitored after 7 days (Figure 9a), which was due to the degradation of the composite. Beyond 14 days of immersion (Figure 9b), the degradation process continued, and the porous degraded surface covered by the NaCl layer was exposed.

**Figure 10:**
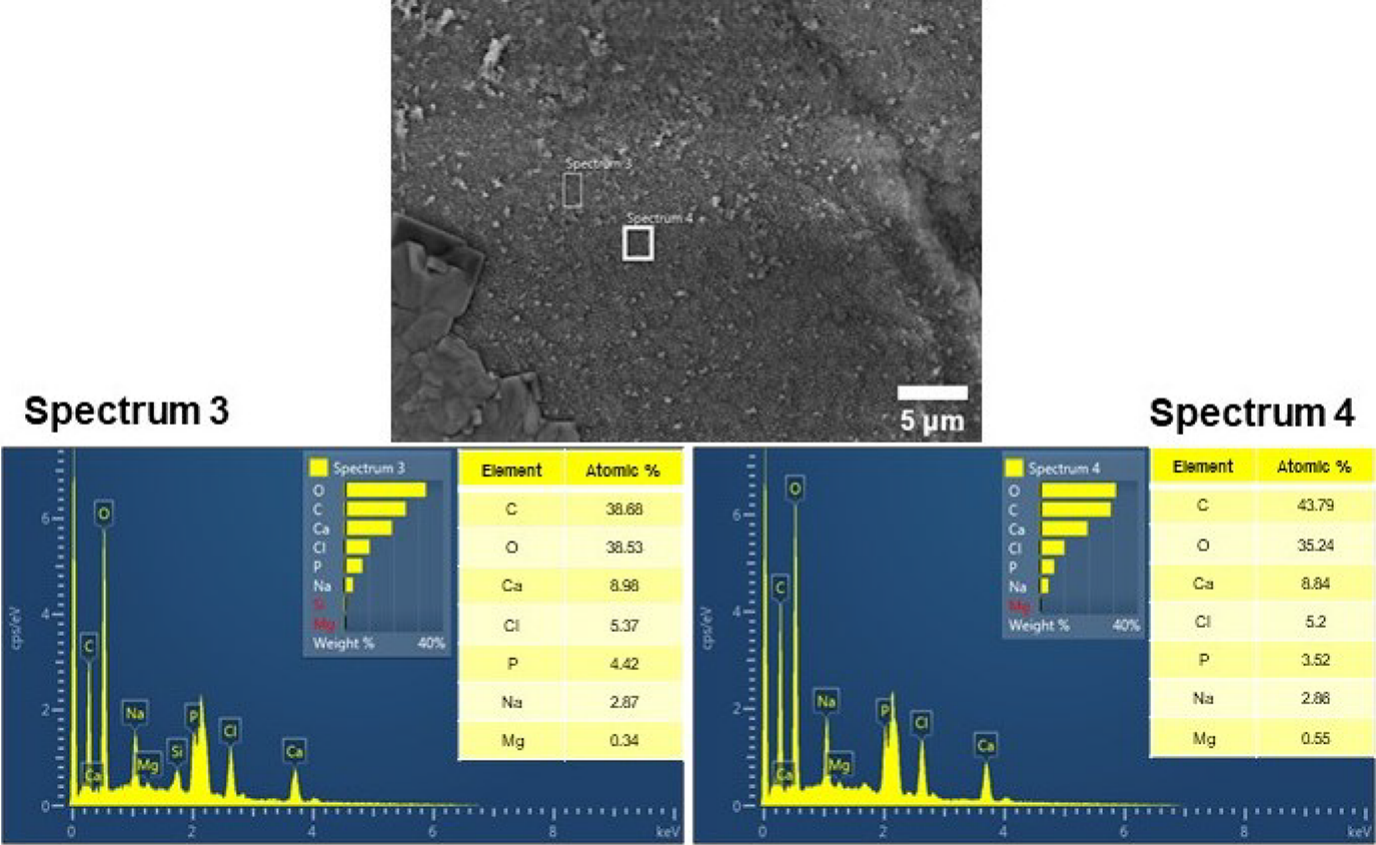
Chemical compositions, calculated by EDX–SEM, of two different surface areas of the coating obtained from A after 14 days in the SBF.

Figure 10 shows the chemical composition of two different surface areas of the composite prepared from A after 14 days of immersion in the SBF. The Ca/P ratio appeared to be slightly over 2 (higher than in hydroxyapatite), so there should be some preliminary phosphate phase formation on the surface while the calcium was also coming from the alginate cross-linking. Moreover, EDX confirmed that the crystals were NaCl.

**Figure 11:**
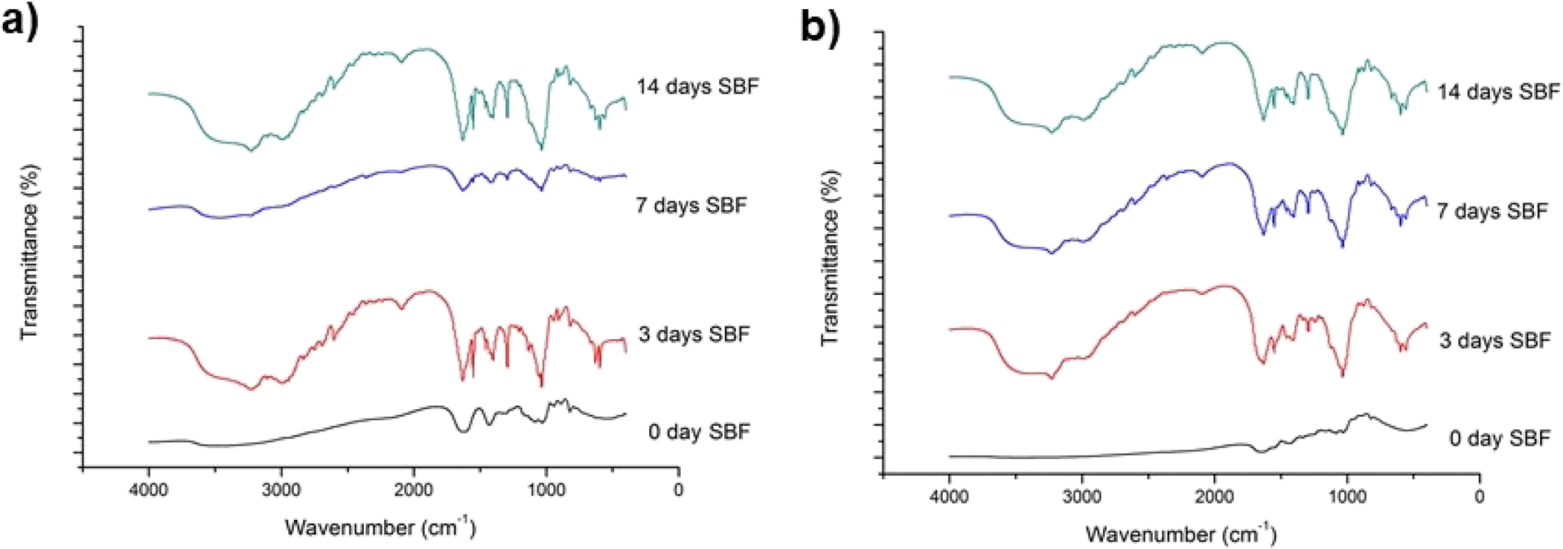
FTIR spectra of both composites, (a) one obtained from A and (b) one prepared from 1A1G, at different immersion times.

In a final attempt to confirm the lack of hydroxyapatite formed on top of the composite coatings, FTIR measurements were taken to search for typical characteristic signals of hydroxyapatite bonds. In both materials, the composite obtained from A (Figure 11a) and the one prepared from 1A1G (Figure 11b), no evolution of the peaks was found with increasing immersion time in the SBF. This fact indicates that there was no ionic interaction typical in the reaction stages to form hydroxyapatite from bioactive glasses.

Therefore, no hydroxyapatite was formed after 14 days in the SBF, although there was evidence of an early stage of hydroxyapatite formation. This effect was probably due to the low content of BG particles in the composite: the content was low, so the particles are embedded in the matrix and have no direct contact with the SBF until the polymer that retains them is degraded. With a longer immersion time in the SBF, the whole mineralization process could be observed. In fact, other experiments conducted by the authors^29,35^ have demonstrated that although the Ca/P ratio was high compared to natural hydroxyapatite after 14 days of immersion, it was reduced and closer to the natural hydroxyapatite value after 21 days. Hydroxyapatite formation after 21 days was proven by SEM, EDX, and XRD. These preliminary studies indicate that after 14 days of immersion, the HA formation could be in the third–fourth stage where a calcium phosphate phase appears as a previous step to the HA crystallization.

### 3.4 Tribomechanical Characterization of the Gelatin Coatings

**Figure 12:**
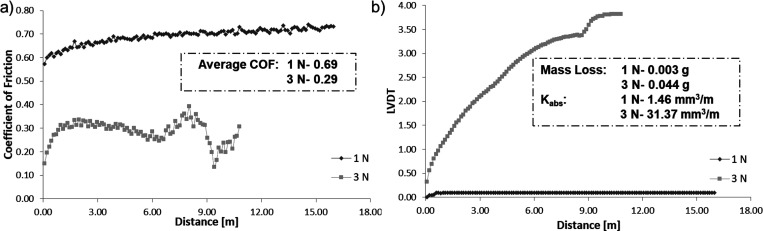
(a) Coefficient of friction and (b) LVDT vs distance of composites, obtained from 1A1G and a 5% BG for an initial spacer size of 355–500 μm and different applied loads of 1 and 3 N. The average COF, mass loss after tribomechanical tests, and absolute wear rates for composites are also presented.

In this work, the effect of the applied load (1 N vs 3 N) on wear behavior was first evaluated. The tribological characterization presented for the composite from 1A1G + 5% BG with a space holder of 355–500 μm (Figure 12) revealed that increasing the load, a higher wear and in turn more mass loss are produced in the gelatin composites. In this way, more severe wear conditions implied a higher associated mass loss. Moreover, the effect of the coating degradation is expected when increasing the applied load.

**Figure 13:**
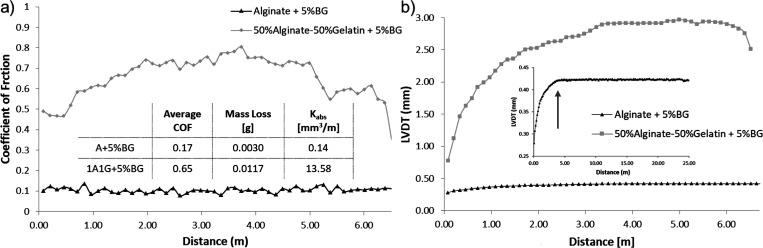
(a) Coefficient of friction and (b) LVDT vs distance of composites obtained from A and 1A1G and 5% BG for a spacer size of 100–200 μm. The average COF, mass loss after tribomechanical tests, and absolute wear rates are also presented.

On the other hand, in Figure 13, the wear resistance of the coating considering one composition, alginate, or the blend, 50 vol % alginate/50 vol % gelatin (both with a 5% BG), is presented for an applied load of 1 N. The linear variable differential transducer (LVDT) versus distance plot is presented in Figure 13b. Observing the slope yield of this curve of the penetration depth as a function of the distance, the wear for the coating from alginate is low and stable. It presents a higher wear resistance in terms of the mass loss. The rolling stage occurs approximately over the 5 m traveled distance (a plateau in the penetration depth is reached). In coating 1A1G, the threshold in the LVDT can be found at around 3 m, with the wear kinetics in this case being much higher than that in the alginate coating (higher slope yield). The wear kinetics is proportional to the mass loss. Moreover, degradation after a 6 m distance is observed, which suggests that the mass loss is higher for composite coating 1A1G. Concerning this composite coating, it is also important to note the significant degree of stress damping, expressed in a greater elastic recovery. This behavior could be very interesting considering the role that this material should play if it intends to replace the functions of the tissue of a joint.

**Figure 14:**
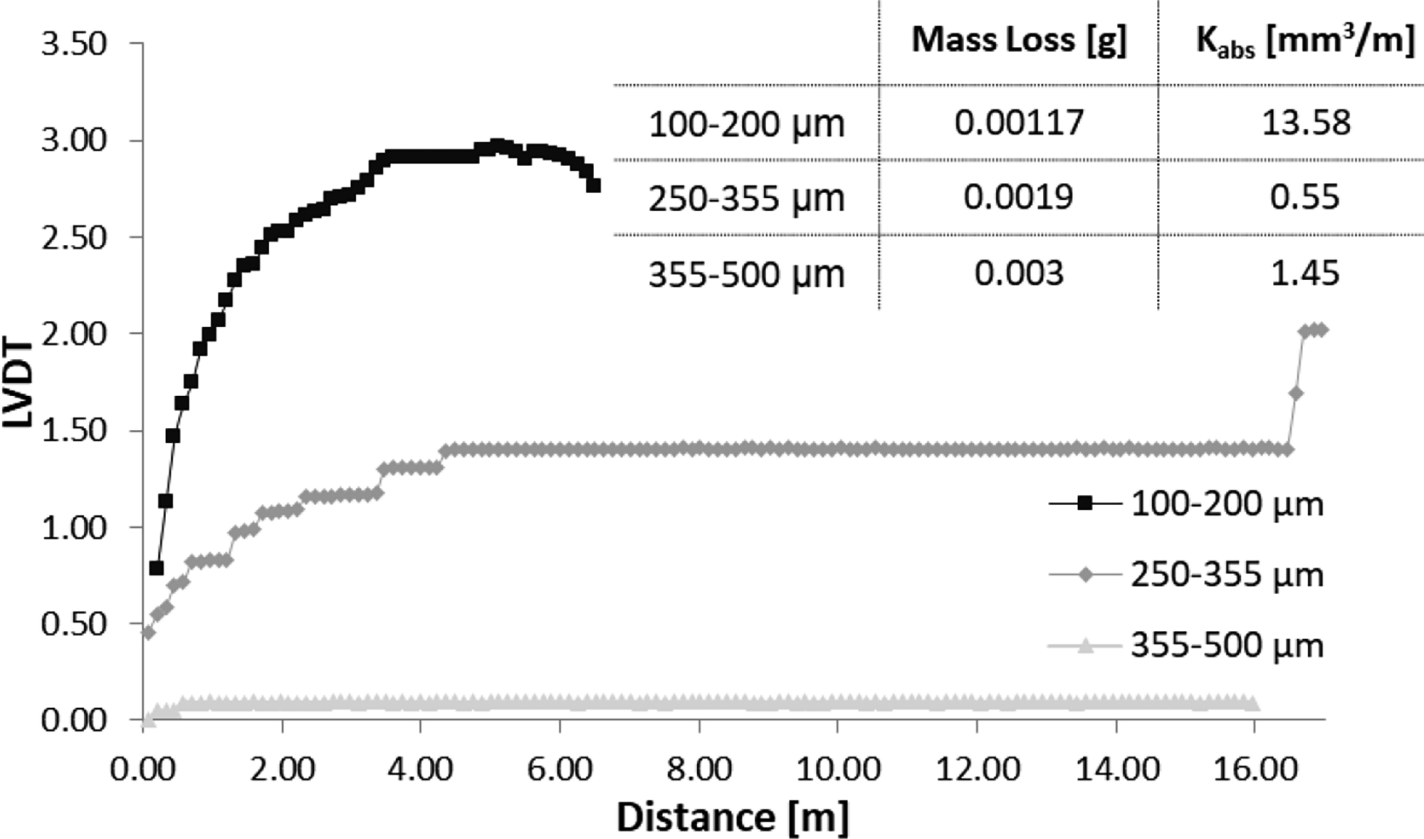
Influence of the pore size of the titanium substrate on the wear behavior (LVDT vs distance) of 1A1G + 5% BG. The mass loss after tribomechanical tests and absolute wear rates of composites are also presented.

In the previous figures, Figures 12 and 13, the outcome of the applied load and the gelatin composites’ chemical composition were verified, respectively. Finally, the effect of the type of porous titanium substrate on the wear behavior of the blend 1A1G + 5% BG was studied (Figure 14). For the intermediate size (250–355 μm), an increase in wear resistance in terms of a lower mass loss and absolute wear coefficient can be observed, if compared with the other types of porous titanium substrates. This fact could be related to the better adhesion of gelatin compounds on this type of porous substrate. A stable behavior is observed for 5 m of travel, and the behavior changes sharply after approximately 16 m of distance traveled. This last effect could be associated to the rupture of layers in the gelatin coating. It is also interesting to consider that the “spring” behavior continues to be pronounced, a fact that could be related to the role that the proportion of gelatin plays in this type of coating.

## 4 Conclusions

Porous c.p. titanium substrates manufactured by the space holder technique with 50 vol % NH_4_HCO_3_ and pore sizes in the range of 250–355 μm are the most recommended in terms of guaranteeing a biomechanical balance (Young’s modulus and yield strength) and biofunctionality (allowing bone ingrowth as well as infiltration and adherence of the gelatinous compound onto the porous Ti substrate). In relation to the wear behavior of coatings, a lower mass loss of the 95% alginate + 5% BG coating was confirmed. However, a higher elastic recovery was observed at the end of the wear tests of the compound incorporating gelatin. This last result is very interesting since this type of coating could replicate the functions of the soft tissue in areas of the joints. In this global scenario, the proposed system (porous substrate coating) has a potential application in the combined replacement/regeneration of hard (cortical bone) and soft (cartilage and skin) tissues (tissue interfaces).
